# Normal Levels of *Sox9* Expression in the Developing Mouse Testis Depend on the TES/TESCO Enhancer, but This Does Not Act Alone

**DOI:** 10.1371/journal.pgen.1006520

**Published:** 2017-01-03

**Authors:** Nitzan Gonen, Alexander Quinn, Helen C. O’Neill, Peter Koopman, Robin Lovell-Badge

**Affiliations:** 1 The Francis Crick Institute, Midland Road, London, United Kingdom; 2 Institute for Molecular Bioscience, The University of Queensland, Brisbane, Queensland, Australia; Duke University Medical Center, UNITED STATES

## Abstract

During mouse sex determination, transient expression of the Y-linked gene *Sry* up-regulates its direct target gene *Sox9*, via a 3.2 kb testis specific enhancer of *S**ox9* (TES), which includes a core 1.4 kb element, TESCO. SOX9 activity leads to differentiation of Sertoli cells, rather than granulosa cells from the bipotential supporting cell precursor lineage. Here, we present functional analysis of TES/TESCO, using CRISPR/Cas9 genome editing in mice. Deletion of TESCO or TES reduced *Sox9* expression levels in XY fetal gonads to 60 or 45% respectively relative to wild type gonads, and reduced expression of the SOX9 target *Amh*. Although human patients heterozygous for null mutations in *SOX9*, which are assumed to have 50% of normal expression, often show XY female sex reversal, mice deleted for one copy of *Sox9* do not. Consistent with this, we did not observe sex reversal in either TESCO^-/-^ or TES^-/-^ XY embryos or adult mice. However, embryos carrying both a conditional *Sox9* null allele and the TES deletion developed ovotestes. Quantitative analysis of these revealed levels of 23% expression of *Sox9* compared to wild type, and a significant increase in the expression of the granulosa cell marker *Foxl2*. This indicates that the threshold in mice where sex reversal begins to be seen is about half that of the ~50% levels predicted in humans. Our results demonstrate that TES/TESCO is a crucial enhancer regulating *Sox9* expression in the gonad, but point to the existence of additional enhancers that act redundantly.

## Author Summary

SOX9, a member of the SOX family of developmental transcription factors related to the Y-chromosomal sex-determining factor SRY, plays pivotal roles in cell differentiation in a variety of developmental contexts including formation of the testes, skeleton, brain, skin, pancreas, gut and kidneys. During mammalian male sex determination, *Sox9* is the critical effector gene through which SRY directs differentiation of Sertoli cells and hence drives testis formation; structural mutation or deletion of *Sox9* causes XY sex reversal in humans and mice. Despite its importance, how *Sox9* is regulated in time and location is poorly understood. Previous studies identified an enhancer element, TES, containing a core element, TESCO, either of which direct reporter gene expression to the developing testis in transgenic mice. However, no loss-of-function mutations have been identified in humans or created in mice to date. Here, we delete TES and TESCO in mice using CRISPR/Cas9 gene editing technology. As a result, *Sox9* expression levels in fetal XY gonads were reduced by ~50%, but no sex reversal occurred. Our results confirm that intact TES/TESCO is required for directing appropriate *Sox9* expression levels in the testis, and highlight the presence of additional enhancers that remain to be identified.

## Introduction

Cell fate choices during development often depend on the expression of key genes that must themselves be regulated in a controlled manner by the action of transcription factors at critical cis-regulatory regions or enhancers. Some of these genes play major roles in multiple cell types and stages, as evidenced by the effects of loss-of-function mutations. Both how they are regulated in a tissue specific manner and how they in turn exert their actions on different sets of target genes depend on the precise cellular context, including the combination of both partner factors and antagonists or competitors, the activity of signaling pathways, and chromatin states [1]. But while particular combinations of transcription factors are required to generate specificity, some appear to be rate limiting. This might also be true of the enhancers that integrate this regulatory input to expression in a particular tissue.

The regulation and action of *Sox9* during sex determination and testis differentiation illustrates many of these issues. SOX9 is a key transcriptional activator required for the specification, differentiation and maintenance of multiple cell types during development and for several stem and progenitor cells in adults [2–7]. Loss-of-function mutations in the gene have pleiotropic effects and levels of expression are critical. In humans, heterozygosity for null mutations affecting protein function, or for translocations that disrupt gene expression, lead to campomelic dysplasia (CD, OMIM 114290; [8]), a severe syndrome, invariably fatal within a few years of birth, with notable defects not only in cartilage development, but also in a range of other tissues including the CNS, gut, kidneys, and sensory systems [7, 9, 10]. In addition, about 70% of XY CD patients show male-to-female sex reversal, indicating that 50% of normal levels of SOX9 is at a threshold for testis differentiation in humans [11, 12]. Heterozygous null mutant mice also show a range of defects in many of the same tissues, and die after birth due to skeletal problems that compromise breathing [9, 13, 14]. In contrast to humans, heterozygosity for a null mutation in *Sox9* in mice does not lead to XY female sex reversal [14, 15]. Homozygous *Sox9*-null mutant mouse embryos die from cardiac neural crest defects at around 11.5 days *post coitum* (dpc), about 24 hours prior to the overt differentiation of testes in XY embryos. However, isolating the XY gonads at 11.5 dpc and placing them in an organ culture system revealed that they began to differentiate as ovaries rather than as testes [14]. Moreover, homozygous conditional deletion of *Sox9* using an early acting *Sf1*-Cre driver was shown to give complete XY sex reversal [15, 16].

The trigger for the early gonad to differentiate as a testis rather than an ovary is the transient expression of the Y-linked sex-determining gene, *Sry* [17–22]. Both the *Sry* gene and its protein product show little conservation between mammalian species apart from an HMG box type of DNA binding domain. Nevertheless, current evidence (reviewed by [23]) suggests that SRY proteins act as weak transcriptional activators within the supporting cell precursor lineage in the XY genital ridge, where their most critical role is to upregulate the expression of *Sox9*.

*Sox9* is transcribed at low levels in both XX and XY genital ridges from about 10.5 dpc [24, 25]. *Sry* expression in XY supporting cell precursors then sharply upregulates *Sox9* expression in XY genital ridges [19, 22, 26, 27]. Once SOX9 reaches a critical threshold, several autoregulatory loops are recruited, including SOX9 acting on its own transcription, to further upregulate its expression and trigger the differentiation of the cell lineage into Sertoli cells. In the absence of *Sry* in an XX gonad, *Sox9* expression is repressed by the action of several ovary-determining and/or anti-testis genes, including components of the WNT-signaling pathway; cells of the supporting cell lineage differentiate into granulosa cells, and an ovary develops (reviewed by [4, 28]). The expression of these anti-testis factors may well account for the time-sensitivity of SRY action; if its expression is delayed by just a few hours, *Sox9* can no longer be upregulated and ovaries develop [29]. Similarly, ectopic activation of the WNT pathway via expression of an activated form of β-catenin leads to XY female sex reversal, although this reflects a failure to maintain rather than initially upregulate *Sox9* [30].

The critical role for SOX9 in testis differentiation is also revealed by gain-of-function studies. XX male sex reversal was found in a human patient with a duplication of the *Sox9* gene [31]. In mice, XX male sex reversal occurs in *Wt1*–*Sox9* transgenic mice [32] and in the *Odd Sex* (*Ods*) transgenic insertional mutant [33, 34], both of which constitutively express *Sox9* in the early gonad irrespective of chromosomal sex. Moreover, when *Ods* is bred onto XY mice carrying an *Sry* deletion, the resulting males are fertile, suggesting not only that SOX9 can substitute for SRY in sex determination, but that it is probably the only critical target gene of SRY [35].

Because *Sox9* is expressed in many tissues, it is not surprising that it has a very complex regulatory region. Indeed, it is thought that this extends about 2 Mb upstream, in a region characterized as a gene desert [36]. The first attempt to dissect and reveal the gonad-specific regulatory regions of *Sox9* began with a mouse *Sox9* bacterial artificial chromosome (BAC) clone (-70 to +50 kb) carrying a *lacZ* reporter gene to generate transgenic mice. Expression of the reporter replicated the endogenous *Sox9* expression pattern in the developing gonads, as well as in some, but not all other sites [37]. *LacZ* expression was first detected in both XX and XY genital ridges around 10.5 dpc, increased by 11.5 dpc, particularly in XY genital ridges, and then became restricted to the testis from about 12.5 dpc, when it became localized to Sertoli cells.

**Fig 1:**
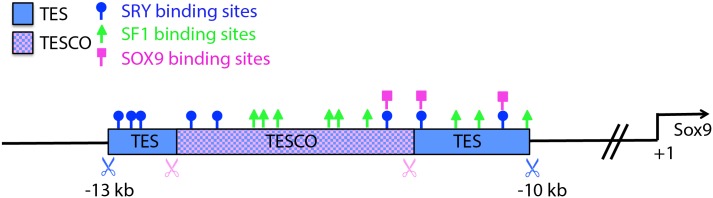
Schematic representation of TES and TESCO. TES (3161 bp, blue box) is located 10–13 kb upstream of the *Sox9* transcription start site. TESCO is located within TES (1293 bp, purple box). The location of binding sites for SF1, SRY and SOX9 transcription factors are indicated. Nine putative sites for SF1 (Green triangular), eight for SRY (blue circle) and three for SOX9 (Pink square) are present.

A reiterative process testing the ability of progressively smaller regions of the BAC to drive reporter gene expression (*LacZ* or *eGFP*) in transgenic mice eventually revealed a 3.2 kb fragment, located between -13 kb to -10 kb upstream of the *Sox9* transcriptional start site, to be sufficient to mimic the gonad-specific expression of *Sox9*. This element was termed TES (for Testis-specific Enhancer of *Sox9*; [37]). Chromatin immunoprecipitation (ChIP) assays showed that SRY and steroidogenic factor 1 (SF1, encoded by the gene *Nr5a1*) directly bind several sites within TES *in vivo* at 11.5 dpc. SOX9 was also shown to be bound to TES at 13.5 dpc, where it is likely to replace SRY in the interaction with SF1 to regulate its own expression in the testis (see Fig 1, S1 Fig and [37]). In support of this, genetic studies indicated that both SRY and SOX9 contributed to reporter gene expression driven by TES. Furthermore, a more highly conserved element of 1.4 kb was further refined within TES and termed TESCO (for TES core element). Mutating the three SRY/SOX9 and the six SF1 binding sites in TESCO abolished the enhancer activity in co-transfection assays *in vitro* and in transgenic mice [37]. TES and TESCO also contain putative binding sites for several other transcription factors known to have roles in sex determination or to maintain supporting cell fate. For example, FOXL2, together with estrogen receptors, are required to maintain granulosa cell fate, such that conditional deletion of *Foxl2* from the adult mouse ovary leads to the expression of SOX9 and transdifferentiation of granulosa cells into Sertoli-like cells [38]. FOXL2 was found to bind TES and to repress *Sox9* transcription *in vivo* and *in vitro*. All of the above suggests that TES/TESCO plays a major role in integrating both positive (i.e. transcriptional activator) and negative (i.e. repressor) effects on *Sox9* transcription during gonad development.

To determine the extent to which TES or TESCO are required for *Sox9* expression in the developing gonad in mice, and if this enhancer is indeed rate limiting for testis differentiation, we deleted TES/TESCO using CRISPR/Cas9 genome editing. Homozygous deletion of either TESCO or TES led to a reduction of *Sox9* expression in the XY gonad, to approximately 60% or 45% of levels seen in wild type controls (respectively), indicating that TES/TESCO is crucial for, but not the sole element involved in, regulating *Sox9* transcription levels during sex determination.

## Results

### Testis development appears normal after deletion of TESCO

To delete TESCO, zygotes were injected with Cas9 mRNA and a pair of sgRNAs, targeting either side of the TESCO sequence (Fig 1, Table 1 and S1 Fig). A total of 26 mice were born after zygote injections of which seven appeared to be mosaic or heterozygous for insertions or deletions at the targeted locus based on PCR analysis. Two males and one female appeared to have the correct sized deletion; the two males were test-bred with C57BL/6J females to confirm transmission of the deleted allele. Sequencing of heterozygous offspring from these matings confirmed a precise 1260 bp deletion within TESCO between the two-targeted Cas9 cleavage sites in both cases; offspring of one of the males carried an additional unrecognised sequence of 11 bp inserted at the deletion site. The 1260 bp deletion included 1252 bp (97%) of the 1282 bp TESCO element. Stable lines were established from these heterozygous offspring. None of the lines showed overt signs of sex reversal in either heterozygous or homozygous TESCO-deleted XY or XX embryos or liveborn mice, and the breeding behavior and fertility of adults appeared normal.

**Table 1:** Primers for CRISPR genome editing.

Application	Primer name	Location	Sequence 5' to 3' (20-mer sgRNA recognition sequences in bold)
TESCO 5’ sgRNA preparation	TESCO-dist-F	Cas9 cleavage target site 41 bp 3' to the 5' end of TESCO	CACC**GTTGGAGTTCCGATTTAGAC**
	TESCO-dist-R		AAACGTCTAAATCGGAACTCCAAC
	TESCO-dist-F-T7		TAATACGACTCACTATAGGG**GTTGGAGTTCCGATTTAGAC**
TESCO 5’ sgRNA preparation	TESCO-prox-F	Cas9 cleavage target site 8 bp 3’ to 3’ end of TESCO	CACCG**TCTGTTAACGTATGACACGA**
	TESCO-prox-R		AAACTCGTGTCATACGTTAACAGAC
	TESCO-prox-F-T7		TAATACGACTCACTATAGGGG**TCTGTTAACGTATGACACGA**
sgRNA preparation	sgRNA-uni.R	pX330 plasmid	AAAAGCACCGACTCGGTGCC
TES 5’ sgRNA preparation	TES-dist-F	Cas9 cleavage target site 25 bp 5' to the 5' end of TES	CACC**GAGGTGTGTGGAAGCGGGCA**
	TES-dist-R		AAACTGCCCGCTTCCACACACCTC
TES 3’ sgRNA preparation	TES prox-F	Cas9 cleavage target site 20 bp 3’ to 3’ end of TES	CACC**GGGAACATTGGCAGGGCCCT**
	TES-prox-R		AAACAGGGCCCTGCCAATGTTCCC

**Fig 2:**
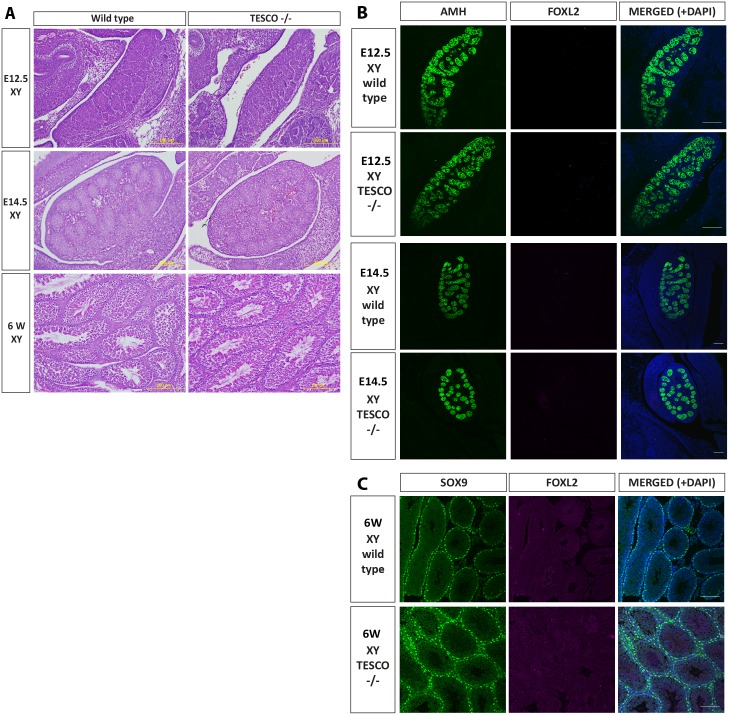
Histological and immunofluorescence analysis of TESCO deleted XY gonads. (A) Haematoxylin and eosin staining of 12.5 dpc, 14.5 dpc and 6 week postnatal XY testis of wild type and TESCO^-/-^ mice. (B) Immunostaining of 12.5 and 14.5 dpc XY testis of wild type and TESCO^-/-^ embryos. Testes were stained for AMH (green), FOXL2 (cyan) and DAPI (blue). (C) Immunostaining of 6 week-old XY testes of wild type and TESCO^-/-^ mice. Testes were stained for SOX9 (green), FOXL2 (cyan) and DAPI (blue).

To look for more subtle phenotypes, gonadal histology and marker expression were studied in detail in one TESCO^-/-^ line at three time points: at 12.5 dpc, when testis cords and the testis-specific coelomic blood vessel are usually apparent and *Sox9* expression levels have reached their peak, while in XX gonads ovarian patterns of gene expression are being established, including the onset of *Foxl2* expression (see, for example: [39]); at 14.5 dpc; and at 6 weeks postnatal. The gonads of TESCO^-/-^ XY and XX embryos and adults at these three stages showed no histological signs of dysmorphology, sex reversal or defective gametogenesis (Fig 2A, S2A Fig).

To study the phenotype at the molecular level, we used immunofluorescence for the embryonic testis marker AMH and ovary marker FOXL2 (Fig 2B) [40, 41]. Both 12.5 dpc and 14.5 dpc TESCO^-/-^ XY gonads were indistinguishable from wild type testes, with AMH expression seen clearly within testis cords at embryonic stages, SOX9 in adult Sertoli cells and no detectable FOXL2 expression (Fig 2B and 2C). Immunofluorescence on sectioned TESCO^-/-^ XX gonads also revealed normal ovarian morphology and FOXL2 expression, and no signs of testis marker expression at embryonic (AMH; S2B Fig) or adult stages (SOX9; S2C Fig). Therefore, the TESCO deletion does not seem to affect sex determination or subsequent gonad development or fertility in either chromosomal sex.

### Sex determination occurs in the absence of TES

Although TESCO is highly conserved among mammals [37, 42] and is bound and regulated by SRY, SF1 and SOX9 itself, it is only half the size of the full TES enhancer in mice, which contains additional SRY/SOX9 and SF1 binding sites. We therefore wanted to explore the possibility that deleting the larger enhancer may result in gonadal phenotypes or sex reversal. To delete TES we used two sgRNAs that target each side of TES, transfected them into ES cells with a Cas9 expression plasmid and screened for clones carrying TES deletions. Of 70 clones screened, 14 (20%) exhibited a heterozygous deletion of TES by PCR, which was verified by sequence analysis (S3A Fig). Indels were always present at both sgRNA target sites in alleles that retained TES. Two clones were injected into 8-cell stage embryos. Several male animals with 100% agouti coat color, corresponding to the ES cell genotype (C57BL/6J x CBA), were obtained. These males were bred to C57BL/6J females and the TES deletion was found in approximately 50% of offspring.

**Fig 3:**
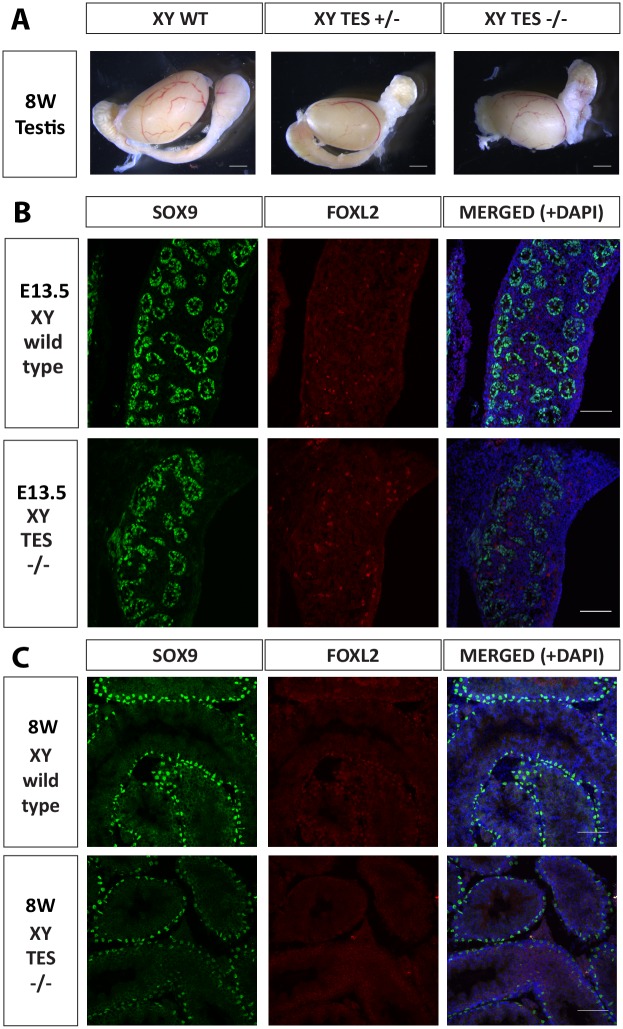
Immunofluorescence analysis of C57BL/6J TES deleted XY gonads. (A) Bright field images of 8-week old wild type, TES^+/-^ and TES^-/-^ testes. (B) Immunostaining of 13.5 dpc XY testis of wild type and TES^-/-^ embryos. (C) Immunostaining of 8-week old XY testes of wild type and TES^-/-^ mice. Testes were stained for SOX9 (green), FOXL2 (red) and DAPI (blue).

We initially analyzed embryos and offspring with a mixed genetic background. There were no obvious signs of sex reversal and as evident in Fig 3A, testis size and morphology were similar between wild type, TES^+/-^ and TES^-/-^ mice. There was also no difference in the weight of testes from 7 month-old mice (S3B Fig). Fertility was examined over a period of 5 months on TES^+/-^ and TES^-/-^ mice, revealing no significant difference in number of litters or of average litter size during this period (S3C and S3D Fig).

C57BL/6J mice are more sensitive than other strains to mutations likely to give XY female development [43]. Genital ridges from C57BL/6J embryos show overall higher levels of expression of genes characteristic of ovary development than those from 129^S8^ [44]. We therefore backcrossed the TES-deleted mice onto the C57BL/6J genetic background for four generations, but still saw no obvious signs of sex reversal. Using immunofluorescence, SOX9 was found to be expressed in XY TES^-/-^ fetal gonads; moreover, no FOXL2 protein could be detected (Fig 3B). Testis cords had formed in the TES deleted XY gonads and they appeared normal (Fig 3B). Testes from 8 week-old TES^-/-^ mice also showed normal seminiferous tubule structure. We noted a slight, consistent reduction in the intensity of SOX9 staining, but stress this is speculative given that immunofluorescence is not a quantitative assay (Fig 3C). Immunofluorescence analysis of TES-deleted XX ovaries revealed normal morphology and expression of FOXL2, and no SOX9 expression (S4 Fig).

### TESCO/TES are required for normal levels of *Sox9* expression in XY gonads

Since deleting either TESCO or TES did not affect sex determination or testis differentiation, we used qRT-PCR to ask whether the deletions had any effect on the levels of expression of *Sox9* or other Sertoli cell markers, notably *Amh*, a direct target gene of SOX9 [45, 46], and *Sox8* which is expressed in XY gonads from 12.5 dpc and acts redundantly with *Sox9* [14, 47–49]. We also examined the granulosa cell markers *Foxl2* and *Wnt4*.

**Fig 4:**
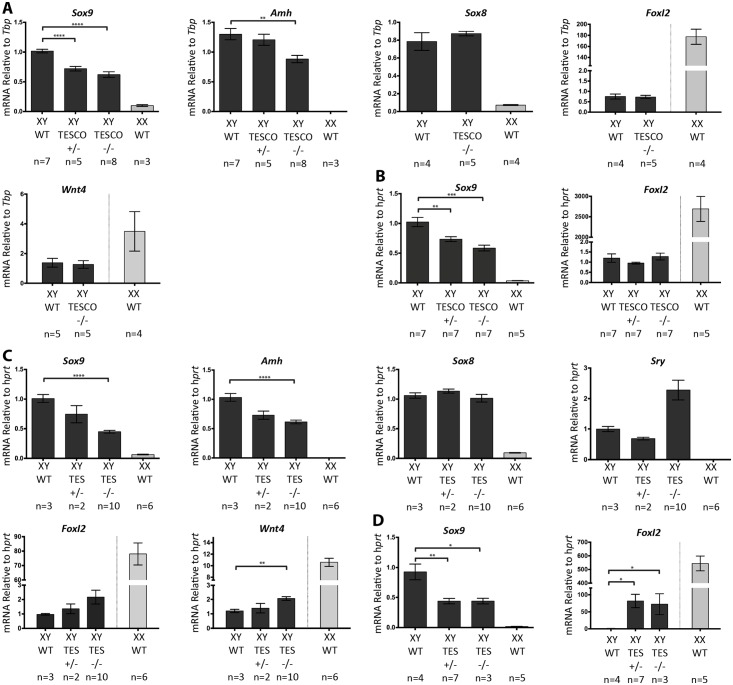
Real-time quantitative RT-PCR of genes involved in male and female sex determination in XY TESCO and TES deleted mice. (A) Gene expression in XY TESCO deleted gonads at 14.5 dpc (B) Gene expression in XY TESCO deleted gonads at 6 weeks (C) Gene expression in XY C57BL/6J TES deleted gonads at 13.5 dpc (D) Gene expression in XY C57BL/6J TES deleted gonads at 8 weeks. Data are presented as mean 2^-ΔΔCt^ values, normalized to *Tbp*/ *Hprt* at the embryonic stages and *Hprt* at the adult stages. Sample size represents number of individuals and is indicated below each genotype. Error bars show SEM of 2^-ΔΔCt^ values. P value is presented above the relevant bars (unpaired, two-tailed t-test on 2^-ΔΔCt^ values). Dark grey bars: XY; light grey bars: XX.

At 14.5 dpc, XY TESCO^+/-^ and TESCO^-/-^ gonads expressed *Sox9* mRNA at around 72% and 62% of wild type levels, respectively (both at p<0.0001; Fig 4A). *Amh* mRNA levels were also reduced in the mutant testes, with TESCO^-/-^ showing 67% of wild type levels (p = 0.002). In contrast, *Sox8*, *Foxl2* and *Wnt4* mRNA levels were unchanged in mutant versus wild type XY gonads at 14.5 dpc (Fig 4A). Gonads from six week-old XY mice gave similar results, with TESCO^+/-^ having 73% (p = 0.006) and TESCO^-/-^ 58% (p = 0.0004) of wild type *Sox9* mRNA levels (Fig 4B). *Foxl2* levels were unchanged in the TESCO-deleted XY gonads (Fig 4B). Analysing gonad-mesonephros pairs at 12.5 dpc also revealed a decrease in *Sox9* mRNA levels with XY TESCO^+/-^ and TESCO^-/-^ gonads showing approximately 81% and 73% of wild type levels, respectively (S5A Fig). The decrease is less than at later stages, but this may be due to “contaminating” *Sox9* expression in the mesonephros. *Foxl2* mRNA levels were also unaffected at this stage (S5A Fig).

These results clearly indicate that the TESCO enhancer is an important regulator of *Sox9* expression throughout testis development, accounting for around 40% of its expression levels.

Since there are additional putative transcription factor binding sites in TES that are not present in TESCO, including several for SF1, SRY and SOX9 (Fig 1 and S1 Fig), we hypothesized that deleting the larger TES enhancer may result in a further decrease in *Sox9* mRNA levels. Consistent with this, qRT-PCR assays on RNA from XY gonads at 13.5 dpc revealed that TES^+/-^ expressed 75% and TES^-/-^ 44% of the wild type levels of *Sox9* mRNA (p<0.0001; Fig 4C). XY TES^-/-^ gonads also had 61% of the wild type levels of *Amh* mRNA (P<0.0001; Fig 4C), while *Sox8* mRNA levels were unchanged.

*Sry* expression is usually downregulated as soon as SOX9 levels increase in supporting cell precursors [20]. This suggests that SOX9 directly or indirectly represses *Sry*. We therefore examined *Sry* mRNA levels, finding them to be 2-fold higher in TES^-/-^ compared to wild type at 13.5 dpc (Fig 4C). However, the difference was not statistically significant, perhaps due to the low levels of *Sry* transcripts at this stage and considerable variability of precise stage between embryos. Similarly, there appeared to be a 2-fold increase in *Foxl2* mRNA levels in TES^-/-^ compared to controls, but again this was not statistically significant and it represents only trace levels compared to expression in XX gonads at 13.5 dpc (Fig 4C). However, a 2-fold increase in *Wnt4* mRNA levels was statistically significant (p = 0.005; Fig 4C). Unlike *Foxl2*, which is usually first expressed only as granulosa cells begin to differentiate, *Wnt4* is expressed equally in XX and XY gonads at early stages, before increasing in ovaries and declining in testes as these develop, but still with robust levels at 13.5 dpc [44].

As in the embryo, the deletion of TES had a greater effect than deletion of TESCO in the adult testis, with TES^+/-^ and TES^-/-^ having 47% of *Sox9* mRNA (p = 0.001 and 0.02 respectively) and 81- and 72-fold increases in *Foxl2* mRNA (p = 0.01) compared to wild type levels (Fig 4D). Even though *Foxl2* expression was increased, it was still far below the level of expression in adult XX ovaries, which is 540-fold that of wild type XY levels. The similar levels of expression seen in the testes of adult heterozygous and homozygous TES mutants is in contrast to the additive effects of each mutant allele seen in the embryo, and in both adult and embryonic TESCO mutants. This could be explained by disruption in TES, but not TESCO mutants, of a positive regulatory feedback loop specific to the adult testis, thereby abrogating the contribution of the remaining copy of TES to *Sox9* transcription. This could involve DMRT1, which is known to be a direct transcriptional activator of *Sox9* in adult Sertoli cells, where it binds sequences both upstream and downstream of the coding region, while expression of SOX9 in granulosa cells leads to upregulation of *Dmrt1* [38, 50].

Assaying mRNA levels of *Sox9* and *Foxl2* in XX gonads carrying TESCO (S5B Fig) or TES deletions (S5C Fig) at embryonic stages did not show any difference in expression compared to wild type ovaries (S5B and S5C Fig).

### Deleting TES on a *Sox8* null background

All three members of the group E Sox transcription factor gene family, *Sox8*, *Sox9* and *Sox10*, are expressed in the developing mouse testis [48, 51–53]. The three proteins are very similar in structure and are likely to be able to regulate overlapping, if not the same, sets of target genes. *Sox8* expression starts at 12.5 dpc, shortly after *Sox9* is upregulated [52]. *Sox8* homozygous null males show no embryonic or early postnatal phenotypes, including in the gonad [48], but seminiferous tubule failure and infertility develop at around 5 months [54].

Although SOX8 function is dispensable with regard to embryonic testis development, several studies have revealed that it has a redundant role with SOX9. When an early-acting and efficient *Sf1-Cre* driver is used with a conditional *Sox9* allele, the result is complete XY female sex reversal [15]. However, if the Cre driver is inefficient and/or acts late, XY female sex reversal or a failure to maintain Sertoli cells is only seen on a *Sox8* null background [14, 49]. This indicates that SOX8 reinforces SOX9 function during testis formation. We therefore sought to determine whether *Sox8* could be a major modifier affecting whether the TES deletion can lead to XY female sex reversal or not.

**Fig 5:**
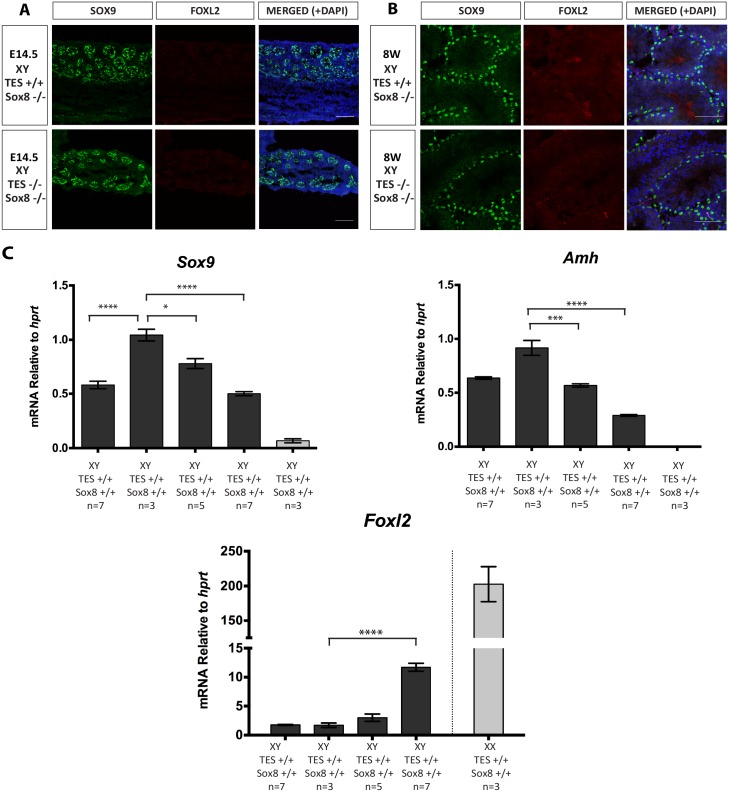
Immunofluorescence and real-time quantitative RT-PCR analysis of mice carrying TES deletion on *Sox8*-null background. (A) Immunostaining of 14.5 dpc XY testis of wild type and TES^-/-^ embryos on a *Sox8*-null background. (B) Immunostaining of 8 week-old XY testes of wild type and TES^-/-^ mice on a *Sox8*-null background. Testes were stained for SOX9 (green), FOXL2 (red) and DAPI (blue). (C) Gene expression in XY TES deleted gonads with *Sox8*-null background at 14.5 dpc. Data are presented as mean 2^-ΔΔCt^ values normalized to *Hprt*. Sample size represents number of individuals and is indicated below each genotype. Error bars show SEM of 2^-ΔΔCt^ values. P value is presented above the relevant bars (unpaired, two-tailed t-test on 2^-ΔΔCt^ values). Dark grey bars: XY; light grey bars: XX.

The TES deletion allele was bred onto a *Sox8* null background and gonads analysed by immunofluorescence at 14.5 dpc (Fig 5A) and 8 weeks (Fig 5B). The XY TES^-/-^; *Sox8*^-/-^ gonads retained SOX9 expression, with no obvious induction of FOXL2, and had normal testis cord or seminiferous tubule morphology. We analysed gonadal gene expression by qRT-PCR at 14.5 dpc, when *Sox8* expression was expected to be maximal. Unexpectedly, *Sox9* mRNA levels were increased by about 2-fold upon deletion of *Sox8* (TES^+/+^; *Sox8*^+/+^ vs. TES^+/+^; *Sox8*^-/-^ p<0.0001, Fig 5C). TES deletion lowered *Sox9* mRNA levels to 77% in TES^+/-^; *Sox8*^-/-^ and to 50% in TES^-/-^; *Sox8*^-/-^ compared to the TES^+/+^; *Sox8*^-/-^ levels (p = 0.01; p<0.0001, respectively, Fig 5C). *Amh* mRNA levels were also significantly reduced, with TES^+/-^; *Sox8*^-/-^ showing 56% (p = 0.0007) and TES^-/-^; *Sox8*^-/-^ 29% (p<0.0001) of the TES^+/+^; *Sox8*^-/-^ *Amh* mRNA levels (Fig 5C). Lastly, *Foxl2* mRNA levels showed a significant increase on a *Sox8* null background (Fig 5C), with an ~11-fold increase in *Foxl2* expression in TES^-/-^; *Sox8*^-/-^ compared to TES^+/+^; *Sox8*^-/-^ or TES^+/+^; *Sox8*^+/+^ (p<0.0001). The above results indicate that the absence of *Sox8* in addition to TES did not exert an additive effect on *Sox9* expression levels nor on the fate of the gonad. However, the additional loss of *Sox8* did lead to higher levels of *Foxl2* compared to the TES deletion on its own (Fig 5C). This suggests that SOX8 as well as SOX9 is normally involved in the repression of *Foxl2* during testis development, supporting their functional redundancy.

### A further reduction in *Sox9* levels leads to development of ovotestes

**Fig 6:**
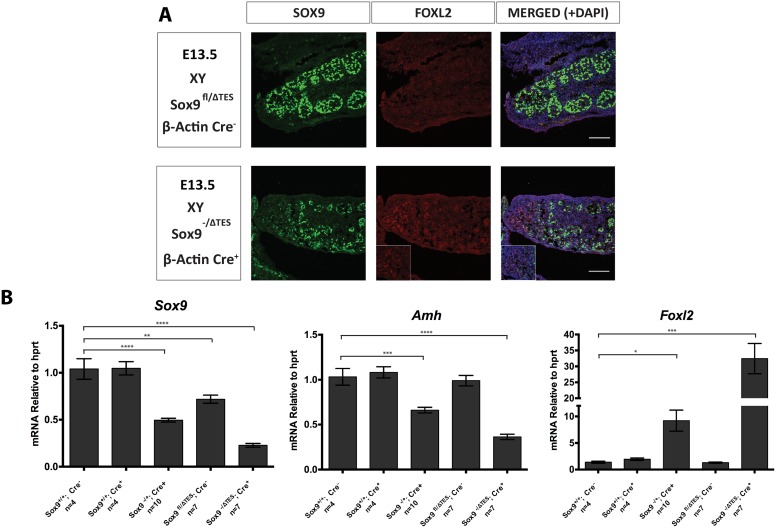
Comparison of *Sox9*^*fl/ΔTES*^ and *Sox9*^*-/ΔTES*^ gonads. (A) Immunostaining of 13.5 dpc XY gonads of *Sox9*^*fl/ΔTES*^ and *Sox9*^*-/ΔTES*^; β-Actin:Cre embryos. Gonads were stained for SOX9 (green), FOXL2 (red) and DAPI (blue). The pole of the gonad is indicated in the inset box. (B) Gene expression in XY gonads with or without conditional *Sox9*-null and TES deletions at 13.5 dpc. Data are presented as mean 2^-ΔΔCt^ values normalized to *Hprt*. Sample size represents number of individuals and is indicated below each genotype. Error bars show SEM of 2^-ΔΔCt^ values. P value is presented above the relevant bars (unpaired, two-tailed t-test on 2^-ΔΔCt^ values).

If about 45% (as in TES^-/-^) or 50% (as in *Sox9*^+/-^) of normal levels of *Sox9* are sufficient to promote testis development in mice, but no *Sox9* (*Sox9*^-/-^) gives complete XY female sex reversal with ovary development, then this raises the question of what level constitutes a minimum threshold level of *Sox9* expression able to direct normal testis development in mice. To address this question, we combined one allele of *Sox9* deleted for TES (*Sox9*^*ΔTES*^*)* with a conditional *Sox9* null mutant allele (*Sox9*^*fl*^) and a ubiquitous Cre driver (β-Actin:Cre). This combination would be predicted to reduce *Sox9* expression in the gonad to about 25% of wild type. Irrespective of genotype, all XY embryos examined at 13.5 dpc appeared to have normal testes except for the embryos that were *Sox9*^*-/ΔTES*^; β-Actin:Cre. All gonads from the latter had a disorganized testis cord structure in the centre and an absence of cords at both poles. Immunofluorescence staining confirmed that they were ovotestes, with SOX9 expressed in the central testicular portion and FOXL2 expression marking the ovarian domains at the poles (Fig 6A). Mice heterozygous for *Sox9-*null mutations die at birth, making it impossible to study the postnatal phenotype and to ask if the ovotestes resolve into testes, ovaries, or stay mixed.

To determine the levels of *Sox9* in the various mutant gonads, and ask if these matched predictions, we performed qRT-PCR assays on RNA from whole XY gonads at 13.5 dpc from the following genotypes: *Sox9*^+/+^ (wild type controls that contain two normal *Sox9* alleles, and which lack the Cre driver; referred to as Cre- in Fig 6B); *Sox9*^+/+^; β-Actin:Cre^+^ (wild type controls that contain two normal alleles of TES and *Sox9* as well as the β-Actin:Cre transgene (referred to as Cre+ in Fig 6B), to rule out any effect of the latter); *Sox9*^-/+^; β-Actin:Cre^+^ (where one *Sox9* allele is wild type, but Cre activity has converted the other floxed allele into a *Sox9* null allele); *Sox9*^*fl/ΔTES*^ (embryos carrying one floxed allele and one TES-deleted allele of *Sox9*, but without the Cre-driver); and *Sox9*^*-/ΔTES*^; β-Actin:Cre^+^ (as before, but with the Cre-driver, resulting in the floxed allele becoming null mutant for *Sox9*). *Sox9* mRNA levels were similar between *Sox9*^+/+^; Cre^-^ and *Sox9*^+/+^; Cre^+^ indicating the β-Actin:Cre transgene has no effect on *Sox9* expression levels. Having one null allele of *Sox9* resulted in 49% of the wild type levels of *Sox9* mRNA (p<0.0001; Fig 6B). *Sox9*^*fl/ΔTES*^; Cre^-^ embryos exhibited 71% of the wild type levels (p<0.01; Fig 6B) (N.B. This is similar to the level found previously for the TES-deletion alone, suggesting that the floxed allele is equivalent to wild type). Finally, having a combination of a *Sox9* null allele and the TES-deleted allele resulted in 23% of wild type levels of *Sox9* mRNA (p<0.0001; Fig 6B).

*Amh* mRNA levels in the same 13.5 dpc testes exhibited a similar trend, with *Sox9*^-/+^; Cre^+^, *Sox9*^*fl/ΔTES*^; Cre^-^, and *Sox9*^*-/ΔTES*^; β-Actin:Cre^+^ having 66% (p = 0.0003), 100% and 36% (p<0.0001) of the wild type levels respectively (Fig 6B). In contrast, *Foxl2* mRNA levels were 9-fold (p = 0.03) higher, unaffected and 32-fold (p = 0.001) higher in the same samples, respectively (Fig 6B).

Because *Foxl2*-positive cells in the ovotestes are not expressing *Sox9* (see Fig 6A), the proportion of cells expressing *Sox9* is reduced compared to controls. It is therefore possible that the level of *Sox9* mRNA per cell is a little higher than 23% of normal. However, given all the other variables, this is still close to the predicted value of about 25%.

Together, these results demonstrate that while 50% of *Sox9* mRNA levels are sufficient to permit testis development in mice, 23% is below a threshold for normal testis development, but above the level at which the XY gonad becomes fully sex reversed.

## Discussion

TES, and its core TESCO, is the only *Sox9* enhancer identified to date giving reporter expression that mimics that of the endogenous gene in the gonads [37]. Moreover, it is able to bind relevant transcription factors and integrate positive and negative control over *Sox9* expression. However, although it is the only enhancer identified, by transgenic assays, within a 120 kb BAC, including 70kb of upstream sequences, it still remained unclear whether it is essential or indeed the only such enhancer. We show here that normal levels of *Sox9* expression in the testis depend on TES/TESCO at both embryonic and adult stages. The TES deletion led to a more pronounced decrease in *Sox9* mRNA levels than the TESCO deletion, which is most likely due to the presence of additional SF1, SRY and SOX9 binding sites in the former. Reduced levels of *Amh* were also seen, consistent with this being an early direct target of SOX9 [45, 46]. However, apart from a small increase in *Foxl2* transcripts, there were no signs of sex reversal in either TES or TESCO homozygous deleted embryos or adults. Moreover, even the former, with only 45% normal levels of *Sox9* mRNA, appeared to have no defects in fertility (S3 Fig).

*Sox9* has perhaps four different phases of expression during gonadal and testis development. First, there is the very low level in the supporting cell precursors prior to the onset of *Sry* expression, and consequently this expression is seen in both XX and XY genital ridges. There is then the phase of upregulation in XY embryos due to the direct effect of SRY acting together with SF1, and the repression seen in XX embryos, presumably due to the activity of anti-testis factors, for example the effect of β-catenin, which may work by inhibiting SF1 activity [55]. There is then a phase characterized by further upregulation and consolidation of high levels of SOX9, which leads to Sertoli cell differentiation. *Sox9* levels are maintained due to several positive feedback loops, including SOX9 acting on itself, as well as FGF9 and prostaglandin D2 [56–59]. In the fetal ovary, it would appear that several factors maintain repression of *Sox9*, including WNT signaling, perhaps FOXL2, and one or more factors yet to be identified [40, 56, 59, 60]. The final phase is seen postnatally, when maintenance of *Sox9* expression in the testis becomes dependent on DMRT1, whereas its repression in the ovary is dependent essentially only on FOXL2 [38, 50, 61–63].

We cannot be certain from the results presented here that TES/TESCO has any role in the expression of *Sox9* during the very early phase. However, assays involving ChIP, TES and TESCO transgenic reporters, and co-transfection *in vitro*, all suggest that both SRY and SF1 do act on the enhancer [37]. Nevertheless, we can conclude that TES cannot be the only enhancer required at these early stages, otherwise TES deletion would have resulted in XY sex reversal.

We can also conclude that levels of expression of *Sox9* at 45% of normal are sufficient for Sertoli cell differentiation and maintenance in the mouse. This was perhaps expected from previous studies looking at mice heterozygous for *Sox9* null mutations, where *Sox9* mRNA levels would likely have been around 50% of normal (as shown here). We found Sertoli cell differentiation even in the absence of *Sox8*, in TES^-/-^; *Sox8*^-/-^ embryos, despite the testes expressing even lower levels of *Amh* and about 11-fold higher levels of *Foxl2* compared to TES^-/-^ embryos (Fig 5C). Although still far from reaching the levels in the ovary (~202-fold compared to TES^+/+^; *Sox8*^-/-^ XX; Fig 5); this level of *Foxl2* may represent the first sign of a shift in the balance from male to female.

We noted that *Sox9* expression was significantly higher in the absence of *Sox8*, which was not commented on by Barrionuevo et al. [49], even though it is apparent in their data. Although *Sox8* expression is known to be dependent on SOX9 [14, 49], this suggests that SOX8 by itself or as heterodimers with SOX9, can repress or interfere with *Sox9* transcription. Perhaps the two proteins have overlapping but not quite identical functions.

Several ovary determining or anti-testis factors have been shown to act on TES/TESCO. They include FOXL2 [38], WNT signaling factors [55], and DAX1 [64], where both the latter seem to act via inhibition of SF1. It was therefore conceivable that deletion of TES or TESCO might lead to signs of testis differentiation in XX gonads. This was not the case, however, as the XX embryos showed normal ovary development and gave rise to fertile females, and we found no evidence of *Sox9* induction (S5B and S5C Fig). This implies that there are other enhancers that can integrate the negative control over *Sox9* transcription by these anti-testis factors, and/or that the upregulation of *Sox9* seen in, for example, *Wnt4* or *Foxl2* mutants, is dependent on transcriptional activators acting on TES/TESCO, which they cannot do if it is deleted [40, 56].

When we combined a conditionally deleted null allele of *Sox9* with a TES deleted allele, which further reduced *Sox9* levels to 23% of normal, XY gonads showed typical signs of ovotestes development, with SOX9 expressed in the central domain, in testis cords that were poorly organized, and FOXL2 expressed in cord-free domains at the poles. Ovotestes often lose one component or the other and resolve into hypoplastic testes or ovaries by birth. Such mice, with smaller than normal testes, can show normal fertility; indeed, having only one functional testis is sufficient [65]. This is probably similar to the situation in humans with CD, where heterozygosity for *SOX9* null mutations leads to XY female sex reversal in the majority of cases. This suggests that the threshold level for *Sox9* is about 25% in mice and 50% in humans.

If TES accounts for only about half the normal levels of *Sox9* expression in the mouse testis, this suggests that there must be one or more additional enhancers responsible for the remainder. The notion of redundant or “shadow” enhancers is now well established (for reviews, see [66, 67]). Indeed, multiple enhancers upstream of *Sox9* have recently been shown to regulate its expression in cartilage [68]. Additional enhancers relevant to the gonad are likely to be outside the 120 kb BAC that was originally screened by transgene reporter assays [37]. Evidence from human patients showing DSD associated with deletions or duplications involving SOX9 flanking regions suggests that these may map at a considerable distance upstream. One region located 516–584 kb upstream of *SOX9*, termed RevSex, may be relevant. This region was originally defined based on four cases of 46,XX *SRY*-negative individuals who presented with testis development. Each patient had a duplication involving a genomic region located ~600 kb upstream of *SOX9* [69–73]. In addition, a 46,XY female with gonadal dysgenesis was reported to harbor a deletion of this region [69]. A recent study further refined this region to about 40 kb in individuals who did not exhibit any other phenotype apart from the sex reversal [74]. A second region implicated in *SOX9* expression in the gonad has been termed XY Sex Reversal (XY SR). This region was recently identified in four 46,XY *SRY*-positive female individuals carrying heterozygous deletions of a minimal 32.5 kb interval located 607.1–639.6 kb upstream [73].

Given that no human case of DSD has yet been described with mutations affecting the TES/TESCO homologous region close to the SOX9 coding region [75], these far upstream regions may have particular importance for levels of *SOX9* expression in the human testis. However, whether these rather large regions contain true enhancers or are involved in chromatin organization or domain structure is not yet known. Nevertheless, there is a degree of conservation between these regions in the human and mouse suggesting that it might be informative to ask if they contain functional enhancers. Therefore, despite the significant contribution made by TES/TESCO, further research is needed in order to gain a comprehensive picture of the array of enhancers that regulate *Sox9* expression in the gonads.

## Materials and Methods

### Design and preparation of sgRNAs and Cas9 mRNA

Two pairs of 20-mer single guide RNAs (sgRNAs) were designed (www.crispr.mit.edu/) to flank and delete either the mouse TESCO (1260 bp deletion, including 1252 bp (97%) of the 1293 bp TESCO sequence) or TES sequences (3194 bp deletion, including the full 3161 bp TES sequence) [37] by CRISPR/Cas9 genome editing. sgRNAs and Cas9 mRNA were prepared as described elsewhere [6]. Briefly, for each sgRNA the complementary pair of oligos (Table 1) was annealed and cloned into pX330 (Addgene #42230). For the TESCO deletion, A T7-sgRNA PCR product was amplified with a T7 promoter sequence introduced on the forward primer in conjunction with a universal reverse primer sgRNA-uni.R (Table 1). This product was used as the template for *in vitro* transcription (IVT) using the MEGAshortscript T7 IVT kit (Life Technologies). The Cas9 coding region was released from pX330 and subcloned into pBluescript II (pBS-Cas9). *Xho*I linearized pBS-Cas9 was used as the template for IVT using the mMESSAGE mMACHINE T7 ULTRA kit (Life Technologies). Both sgRNAs and Cas9 mRNA were purified using the MEGAclear kit (Life Technologies) and eluted in RNase-free water.

### Microinjection of the TESCO sgRNA

30 ng/μl of Cas9 mRNA and 15 ng/μl of each sgRNA diluted in RNase-free water were injected into one pronucleus of C57BL/6J x CBA F1 hybrid one-cell embryos, as described [76, 77]. Injected embryos were cultured overnight to the two-cell stage, and then surgically transferred into oviducts of day-of-plug pseudopregnant CD1 mice.

### Transfection of TES sgRNA

Two pX330 plasmids containing the sgRNAs (Table 1) were transfected along with the pPGK-Puro plasmid (Addgene #11349) to C57BL/6J x CBA F1 hybrid ES cells using Nucleofector according to the manufacturer’s protocol (Mouse ES cells Nucleofector kit, Lonza). ES cells were cultured in standard 2i/LIF media: serum-free media NDiff (Stem Cell Sciences) supplemented with MEK inhibitor PD0325901 (1 μM; Axon Medchem), GSK3 inhibitor CH99021 (3 μM; Axon Medchem) and 1000 U/ml leukaemia inhibitory factor (LIF) (Millipore). Approx. 36 hours post transfection the cells were selected with 2 μg/ml Puromycin (Life Technologies) for 48 hours. Following selection, the ES cells were returned to 2i/LIF media and clones were picked a week later. Single clones were screened for the deletion using PCR spanning the TES deletion site (TES del-F and TES del-R which amplify a 3.5kb band in WT cells and a 327 bp band in TES deleted cells, Table 2). All clones were heterozygous for the deletion. Sequencing was carried out to verify the deletion as well as to examine the undeleted allele, which in all cases contained indel mutations on both sides of TES.

**Table 2:** Primers for genotyping TES/TESCO deleted mice.

Primer name	Description	Sequence 5' to 3'	Product and size
TESCO del-F1	Flanks 5’ end of TESCO	TCTACTCTGAGAAAGCACACACATC	F1+R2: 1775 bp in WT allele, 492 bp in deleted allele
TESCO del-F3	Internal to TESCO	TCCACCAGCATTGGTTCAAG	F3+R2: 545 bp in thw WT allele, no product in deleted allele
TESCO del-R2	Flanks 3’ end of TESCO	TACAAGAAAGACCCAGTTTAGCATTC	
TES del-F	Flanks 5’ end of TES	GGGCTACAGAGTAAGACCATA	F+R: 3521 bp in WT allele, 327 bp in deleted allele
TES Int-F	Internal to TES	GGATAAGTGAATTAGCCAGCT	Int F+R: 208 bp in WT allele, no product in deleted allele
TES del-R	Flanks 3’ end of TES	GGAAGGAGAGACCATCTAC	
Sex-F	X/Y chromosome	GATGATTTGAGTGGAAATGTGAGGTA	McFarlane et al., 2013
Sex-R	X/Y chromosome	CTTATGTTTATAGGCATGCACCATGTA	
ZFY-F	Y chromosome	GACTAGACATGTCTTAACATCTGTCC	Koopman et al., 1991
ZFY-R	Y chromosome	CCTATTGCATGGACAGCAGCTTATG	

### Genetically altered mice

Transgenic founders with the targeted deletion of the TESCO sequence were identified amongst live born mice by PCR genotyping (all genotyping primers are listed in Table 2). All genotyping was performed using genomic DNA extracted from tail tissue of embryos or ear notch tissue of juvenile animals. The primer pair TESCO del-F1 and TESCO del-R2 amplified a single 1775 bp product in wild type animals whereas a shorter product of approximately 492 bp (depending on the exact size of the deletion) was preferentially amplified or co-amplified with the larger product in animals heterozygous or mosaic for the TESCO deletion. Founder males were bred with C57BL/6J females to verify that deleted alleles were transmitted to progeny. The deletions were verified by cloning and sequencing PCR amplified products from heterozygous offspring of founders. Stable lines were established from two founders, both of which bore a precise 1260 bp deletion between the two targeted Cas9 cleavage sites (with one also bearing an 11 bp insertion of unrecognised sequence); only one of these lines was used for subsequent analysis.

For the TES deletion, two lines of ES cells harbouring the TES deletion were injected into 8-cell stage embryos from outbred Parkes female mice, which were then transferred into oviducts of day-of-plug pseudopregnant C57BL/6J x CBA F1 hybrid recipients. Several 100% Agouti pups (“chimeras”) were born. Males were bred to C57BL/6J females to give germline transmission and expand the colony. Genotyping of animals was carried out using the same PCR primer set used to genotype the ES cells (Table 2).

*Sox8*-null mutant mice were kindly provided by Michael Wegner, University of Erlangen, Germany [48]. All other strains, including C57BL/6J and CBA were obtained from colonies maintained within the two institutions.

### Timed mating and tissue preparation

Embryos or offspring homozygous for either the TES or the TESCO deletion were produced by crossing heterozygous animals. Embryos were collected from timed-mating at embryonic day 12.5 dpc, 13.5 dpc or 14.5 dpc, with noon of the day of plug designated as 0.5 dpc. At 12.5 dpc, gonadal sex is usually but not always possible to judge by visual assessment, however by 13.5 dpc and 14.5 dpc testis cords are clearly visible. For postnatal analysis, phenotypic sex was first assessed by inspection of external genital anatomy (including ano-genital distance). Pups were then killed and dissected for inspection of the reproductive tracts and gonads. For all embryos and pups, chromosomal sex was determined by PCR genotyping with primers Sex-F and Sex-R [78] or by PCR for the Y chromosome gene, *Zfy1* [18]. To clearly distinguish between heterozygous and homozygous animals, we employed two complementary PCR reactions: the first to amplify the deleted allele (TESCO del-F1 x TESCO del-R2 for TESCO deletion and TES del-F and TES del-R for TES deletion) while the second amplified the wild type allele (primer pair TESCO del-F3 x TESCO del-R2 for TESCO deletion and TES Int-F x TES del-R for TES deletion, Table 2).

For 12.5 dpc embryos, paired gonad-mesonephros complexes were dissected out, with the mesonephros trimmed to the length of the gonads, and immediately stored in RNAlater RNA stabilization solution (Invitrogen) until required for gene expression analysis by qRT-PCR. For 13.5 dpc or 14.5 dpc embryos, single gonad without mesonephric tissue were collected. For adults at 6 or 8 weeks, an entire ovary or part of a single testis was similarly preserved in RNAlater solution. Whole embryos (12.5 dpc, 13.5 dpc and 14.5 dpc) or whole gonads (6 or 8 weeks postnatal) were also fixed overnight in 4% paraformaldehyde in phosphate-buffered saline (PBS) at 4°C, washed three times with PBS at 4°C, then dehydrated and embedded in paraffin or OCT. The embedded samples were sectioned for immunofluorescence or haematoxylin and eosin staining.

### Quantitative Real-Time Polymerase Chain Reaction (qRT-PCR)

Total RNA was extracted and subjected to DNaseI treatment using either an RNeasy Micro or Mini kit (Qiagen), for fetal and postnatal tissue samples, respectively. RNA yield was quantified with a NanoDrop spectrophotometer (NanoDrop Technologies), and 100–200 ng RNA (12.5 dpc, 13.5 dpc and 14.5 dpc) or 1000–1500 ng RNA (6 and 8 weeks) was used to synthesize cDNA with the High Capacity cDNA Reverse Transcription kit (Applied Biosystems) or the SuperScript^®^ III Reverse Transcriptase (Invitrogen). qRT-PCR reactions were performed in triplicate or quadruplicate using SYBR Green PCR master mix (Invitrogen) and 150 nM each of forward and reverse primers, and analyzed on a Viia7^™^ Real-Time PCR System (Invitrogen) or the Applied Biosystems 7500 Real-Time PCR System (Thermo Fischer Scientific). Primers are listed in Table 3. Relative mRNA levels were determined by calculating 2^-ΔΔCt^ values relative to the normalizer genes Tbp or Hprt (12.5 dpc, 13.5 dpc and 14.5 dpc) or Hprt (6 and 8 weeks). Relative gene expression is presented as the mean 2^-ΔΔCt^ values (Error bars are SEM of the 2^-ΔΔCt^) for multiple single gonads from individual embryos (sample sizes as indicated on charts). Statistical analysis was performed using unpaired, two-tailed t-tests on the 2^-ΔΔCt^ values.

**Table 3:** Primers for Real-time quantitative RT-PCR.

Primer name	Gene	Sequence 5' to 3'
AQ- Sox9-F	*Sox9*	AGTACCCGCATCTGCACAAC
AQ- Sox9-R		TACTTGTAATCGGGGTGGTCT
AQ- Foxl2-F	*Foxl2*	AGGGAGAGAATAAAACATTCATGG
AQ- Foxl2-R		GCAAACTCCAAGGCCATTAC
AQ- Amh-F	*Amh*	CGAGCTCTTGCTGAAGTTCCA
AQ- Amh-R		GAAGTCCACGGTTAGCACCAA
AQ- Sox8-F	*Sox8*	GCAAGACCCTAGGCAAGCTGT
AQ- Sox8-R		TCTGGGTGGTCTTTCTTGTGC
AQ- Wnt4-F	*Wnt4*	CTGGACTCCCTCCCTGTCTTT
AQ-Wnt4-R		CATGCCCTTGTCACTGCAA
AQ- Tbp-F	*Tbp*	ACGGACAACTGCGTTGATTTT
AQ- Tbp-R		ACTTAGCTGGGAAGCCCAAC
AQ- Hprt1-F	*Hprt1*	TCCTCCTCAGACCGCTTT
AQ- Hprt1-R		CCTGGTTCATCATCGCTAATC
NG- Sox9-F	*Sox9*	AAGAAAGACCACCCCGATTACA
NG- Sox9-R		CAGCGCCTTGAAGATAGCATT
NG- Foxl2-F	*Foxl2*	CGGCATCTACCAGTACATCATAGC
NG- Foxl2-R		GCACTCGTTGAGGCTGAGGTTG
NG- Amh-F	*Amh*	CCACGGTTAGCACCAAATAGC
NG- Amh-R		CACACAGAACCTCTGCCCTACTC
NG- Sox8-F	*Sox8*	AGCGAGAAGAGGCCGTTTG
NG- Sox8-R		TCAGTACCAGAGTCTGAGTCG
NG- Sry-F	*Sry*	GCTGGGATGCAGGTGGAAAA
NG- Sry-R		CCCTCCGATGAGGCTGATATT
NG- Hprt1-F	*Hprt1*	GCTTGCTGGTGAAAAGGACCTCTCGAAG
NG- Hprt1-R		CCCTGAAGTACTCATTATAGTCAAGGGCAT

### Haematoxylin and eosin (H&E) and immunofluorescence staining

H&E histological staining was performed on 7μm-thick sagittal sections (for 12.5 dpc, 14.5 dpc and 6 weeks) according to standard protocols. For the experiments on the TESCO deletion, immunofluorescence staining was also performed on 7μm-thick sagittal sections (for TESCO) or 12 μm-thick sections (for TES), as described elsewhere [53], using (as primary and secondary antibodies, respectively): goat anti-AMH (1:500, Santa Cruz Biotechnology) and donkey anti-goat Alexa Fluor 488 (1:200, Invitrogen); mouse anti-SOX9 (1:100, Abnova) and donkey anti-mouse Alexa Fluor 647 (1:200, Invitrogen); rabbit anti-FOXL2 (1:650, generated as described by [53] and donkey anti-rabbit Alexa Fluor 647 (1:200, Invitrogen). For the experiments on the TES deletion, immunofluorescence staining was performed using rabbit anti-SOX9 (1:300, a generous gift from Francis Poulat, Institute of Human Genetics, Montpellier, France) with donkey anti-rabbit Alexa Fluor 488 (1:500, Invitrogen) and goat anti-Foxl2 (1:300, Novus) with donkey anti-goat Alexa Fluor 568 (1:500, Invitrogen). All immunofluorescence slides were also stained with 4',6-diamidino-2-phenylindole (DAPI, Molecular Probes), to visualize nuclear DNA.

### Animal ethics statement

All procedures involving animals and their care conformed to institutional, state and national guidelines or laws. This study was approved by the University of Queensland Animal Ethics Committee and by the UK Home Office (PPL 70/8560).

## Supporting Information

S1 Fig Sequence of TES and TESCO. The sequence of TES (3161 bp) and TESCO within it (1293 bp, pink box) is presented along with the location of the transcription factor binding sites for SF1, SRY and SOX9. Nine putative sites for SF1 (SF1 1–9, in Green), eight for SRY (SRY 1–8, in Orange) and three for SOX9 (SOX9 1–3, in blue) are presented in the filled double arrows. The data were adapted from [37].
(TIF)

S2 Fig Histological and immunofluorescence analysis of TESCO deleted XX gonads. (A) Haematoxylin and eosin staining of 12.5 dpc, 14.5 dpc and 6 week-old XX ovaries of wild type and TESCO^*-/-*^ mice. (B) Immunostaining of 12.5 dpc and 14.5 dpc XX ovaries of wild type and TESCO^*-/-*^ embryos. Ovaries were stained for AMH (green), FOXL2 (cyan) and DAPI (blue). (C) Immunostaining of 6 week-old XX ovaries of wild type and TESCO^*-/-*^ mice. Ovaries were stained for SOX9 (green), FOXL2 (cyan) and DAPI (blue).
(TIF)

S3 Fig Testis weight and fertility test for XY TES deleted mice. (A) Sanger sequencing results and blast to the wild type sequence at the genomic region containing the TES enhancer. The break points are located within the TES 5’ and TES 3’ sgRNAs sequences. (B) Testis weight (in mg) of wild type, TES^+/-^ and TES^-/-^ of mice at 7 months. The weight is presented as an average between the right and left testis of each individual mouse. (C) Fertility test that presents the average number of litters that each TES^+/-^ and TES^-/-^ mouse produced over a period of 5 months. The number of mice tested in each group in represented with the ‘n’ number below the column. (D) Fertility test that presents the average litter size that each TES^+/-^ and TES^-/-^ mouse produced over a period of 5 months. The number of mice tested in each group in represented with the ‘n’ number below the column.
(TIF)

S4 Fig Immunofluorescence analysis of C57BL/6J TES deleted XX gonads. (A) Immunostaining of 13.5 dpc XX ovaries of wild type and TES^-/-^ embryos. (B) Immunostaining of 8 weeks old XX ovaries of wild type and TES^-/-^ mice. Ovaries were stained for SOX9 (green), FOXL2 (red) and DAPI (blue).
(TIF)

S5 Fig Real-time quantitative RT-PCR of genes involved in male and female sex determination in XY and XX TESCO and TES deleted mice. (A) Gene expression in XY TESCO deleted gonads-mesonephros pairs at 12.5 dpc (B) Gene expression in XX TESCO deleted gonads at 14.5 dpc (C) Gene expression in XX TES deleted gonads at 13.5 dpc (D) Gene expression in XX TESCO deleted gonads at 6 weeks (E) Gene expression in XX TES deleted gonads at 8 weeks. Data are presented as mean 2^-ΔΔCt^ values normalized to Tbp/ Hprt. Sample size represents number of individuals and is indicated below each genotype. Error bars show SEM of 2^-ΔΔCt^ values. P value is presented above the relevant bars (unpaired, two-tailed t-test on 2^-ΔΔCt^ values). Dark grey bars: XY; light grey bars: XX.
(TIF)
