# Correction: Normal Levels of *Sox9* Expression in the Developing Mouse Testis Depend on the TES/TESCO Enhancer, but This Does Not Act Alone

**DOI:** 10.1371/journal.pgen.1006584

**Published:** 2017-02-01

**Authors:** Nitzan Gonen, Alexander Quinn, Helen C. O’Neill, Peter Koopman, Robin Lovell-Badge

Errors were introduced during the production process resulting in the incorrect labelling of the genotypes in Fig 5C. Please view the correct Fig 5 here.

**Fig 5:**
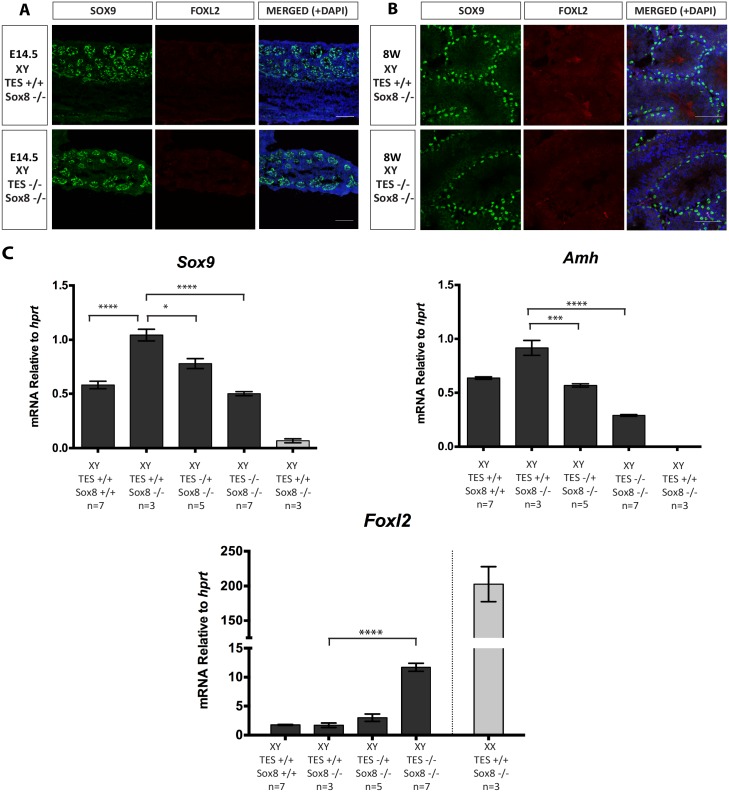
Immunofluorescence and real-time quantitative RT-PCR analysis of mice carrying TES deletion on *Sox8*-null background. (A) Immunostaining of 14.5 dpc XY testis of wild type and TES^-/-^ embryos on a *Sox8*-null background. (B) Immunostaining of 8 week-old XY testes of wild type and TES^-/-^ mice on a *Sox8*-null background. Testes were stained for SOX9 (green), FOXL2 (red) and DAPI (blue). (C) Gene expression in XY TES deleted gonads with *Sox8*-null background at 14.5 dpc. Data are presented as mean 2^-ΔΔCt^ values normalized to *Hprt*. Sample size represents number of individuals and is indicated below each genotype. Error bars show SEM of 2^-ΔΔCt^ values. P value is presented above the relevant bars (unpaired, two-tailed t-test on 2^-ΔΔCt^ values). Dark grey bars: XY; light grey bars: XX.
