# Supplementary figures and images for: Normal Levels of Sox9 Expression in the Developing Mouse Testis Depend on the TES/TESCO Enhancer, but This Does Not Act Alone
Source: PLoS Genet. 2017 Jan 3;13(1):e1006520. doi: 10.1371/journal.pgen.1006520 (PMC5207396; doi:10.1371/journal.pgen.1006520)

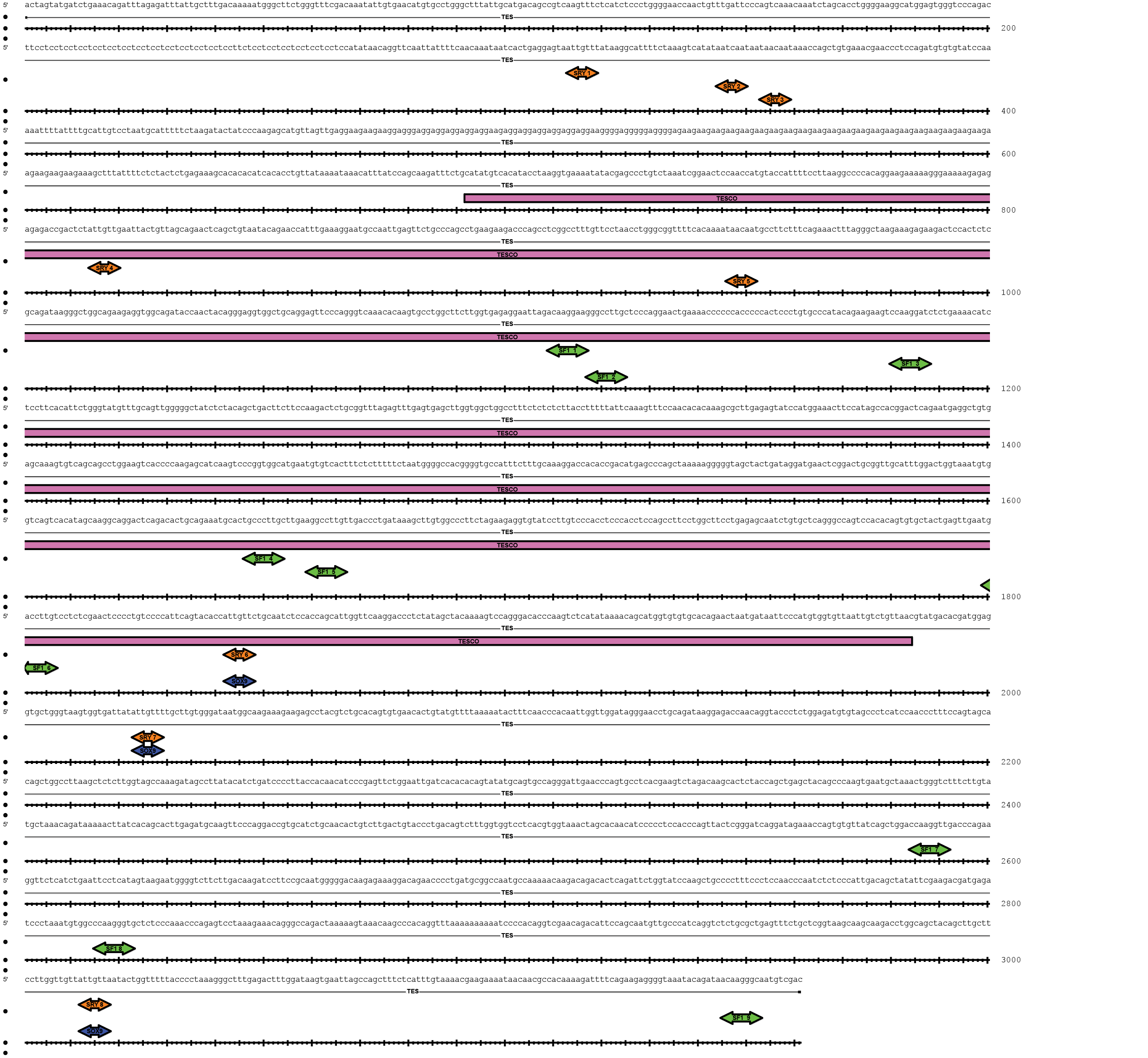

Supplement: S1 Fig — The sequence of TES (3161 bp) and TESCO within it (1293 bp, pink box) is presented along with the location of the transcription factor binding sites for SF1, SRY and SOX9. Nine putative sites for SF1 (SF1 1–9, in Green), eight for SRY (SRY 1–8, in Orange) and three for SOX9 (SOX9 1–3, in blue) are presented in the filled double arrows. The data were adapted from [37]. (TIF) [file pgen.1006520.s001.tif]

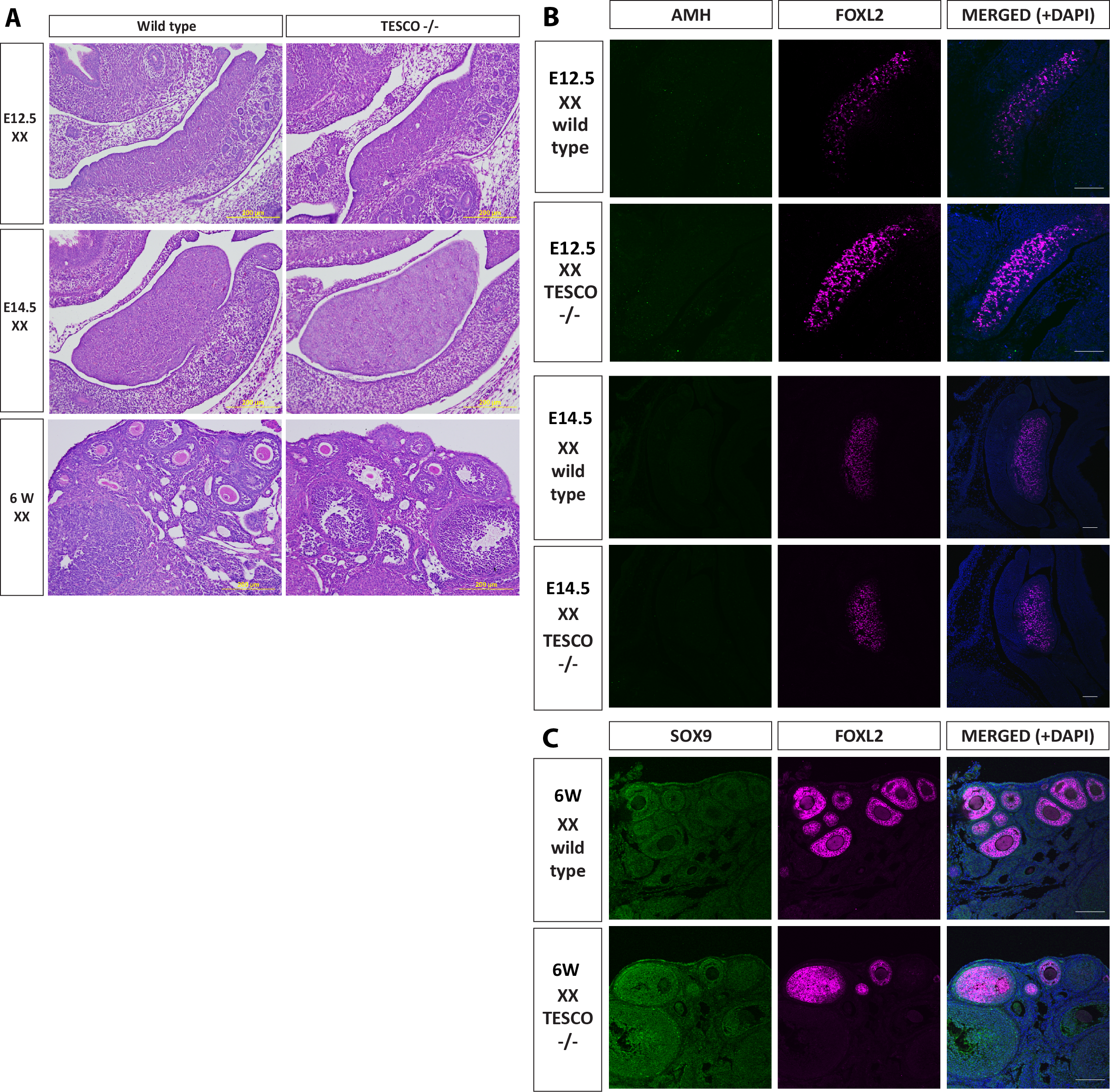

Supplement: S2 Fig — (A) Haematoxylin and eosin staining of 12.5 dpc, 14.5 dpc and 6 week-old XX ovaries of wild type and TESCO-/- mice. (B) Immunostaining of 12.5 dpc and 14.5 dpc XX ovaries of wild type and TESCO-/- embryos. Ovaries were stained for AMH (green), FOXL2 (cyan) and DAPI (blue). (C) Immunostaining of 6 week-old XX ovaries of wild type and TESCO-/- mice. Ovaries were stained for SOX9 (green), FOXL2 (cyan) and DAPI (blue). (TIF) [file pgen.1006520.s002.tif]

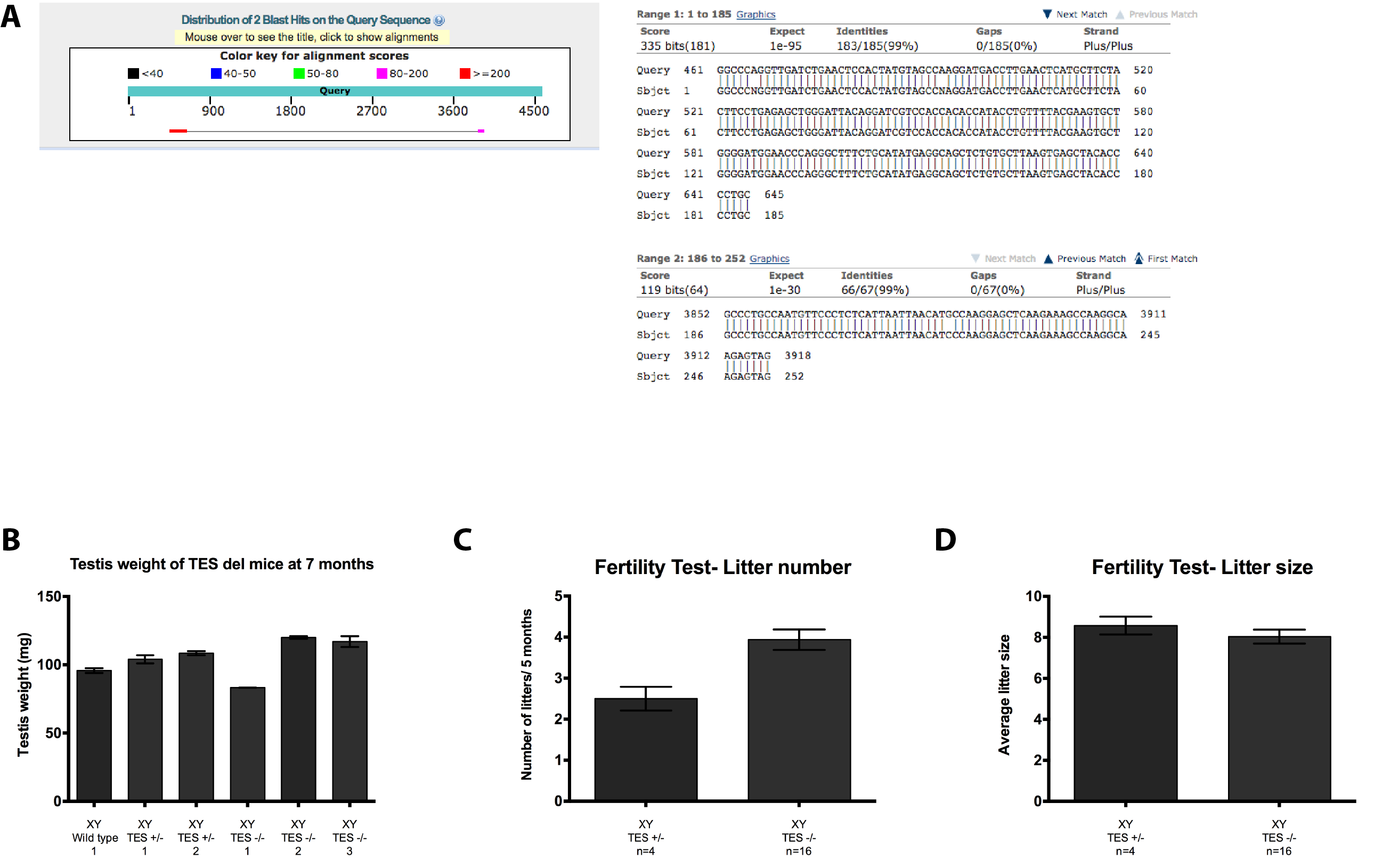

Supplement: S3 Fig — (A) Sanger sequencing results and blast to the wild type sequence at the genomic region containing the TES enhancer. The break points are located within the TES 5’ and TES 3’ sgRNAs sequences. (B) Testis weight (in mg) of wild type, TES+/- and TES-/- of mice at 7 months. The weight is presented as an average between the right and left testis of each individual mouse. (C) Fertility test that presents the average number of litters that each TES+/- and TES-/- mouse produced over a period of 5 months. The number of mice tested in each group in represented with the ‘n’ number below the column. (D) Fertility test that presents the average litter size that each TES+/- and TES-/- mouse produced over a period of 5 months. The number of mice tested in each group in represented with the ‘n’ number below the column. (TIF) [file pgen.1006520.s003.tif]

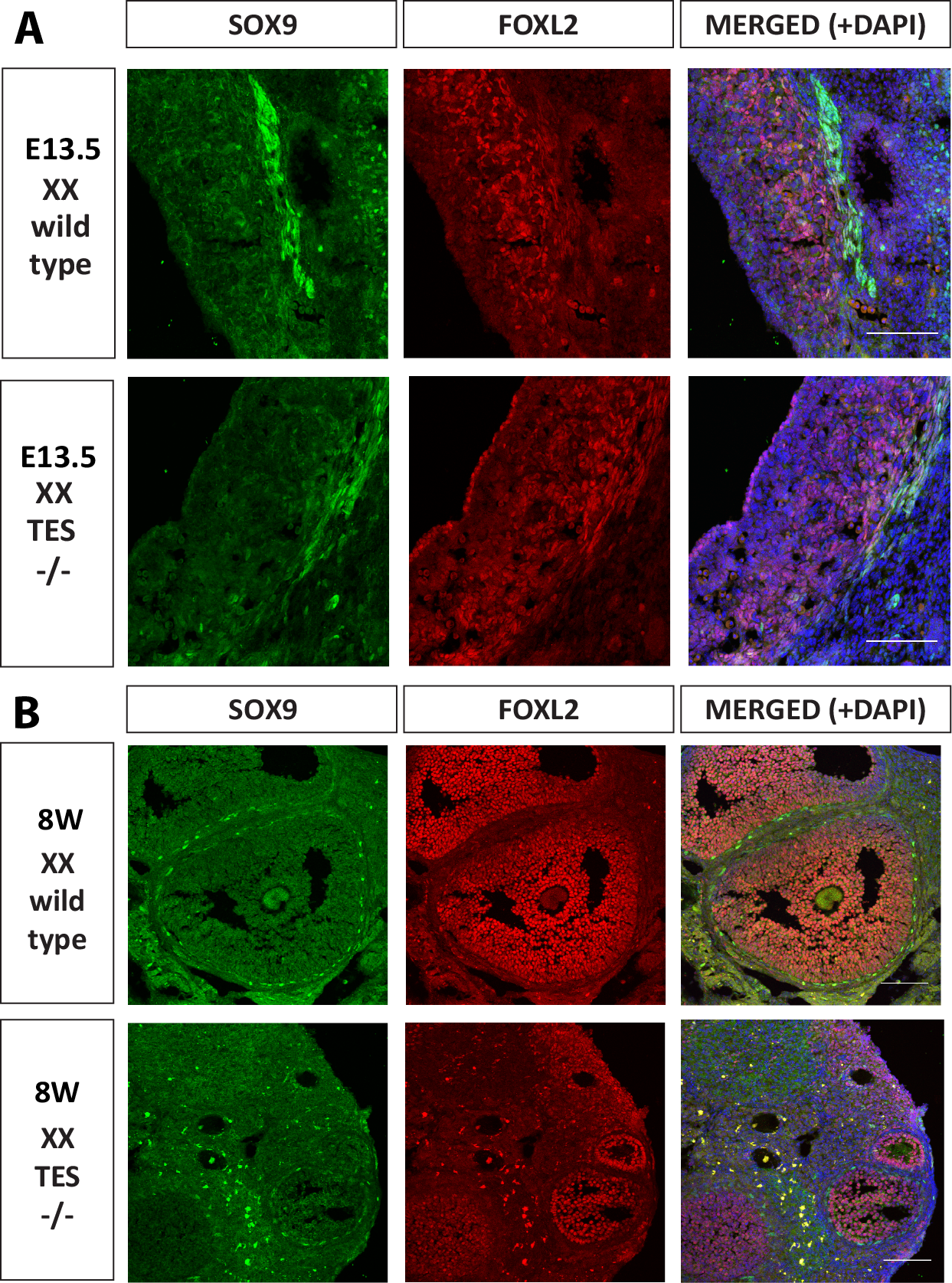

Supplement: S4 Fig — (A) Immunostaining of 13.5 dpc XX ovaries of wild type and TES-/- embryos. (B) Immunostaining of 8 weeks old XX ovaries of wild type and TES-/- mice. Ovaries were stained for SOX9 (green), FOXL2 (red) and DAPI (blue). (TIF) [file pgen.1006520.s004.tif]

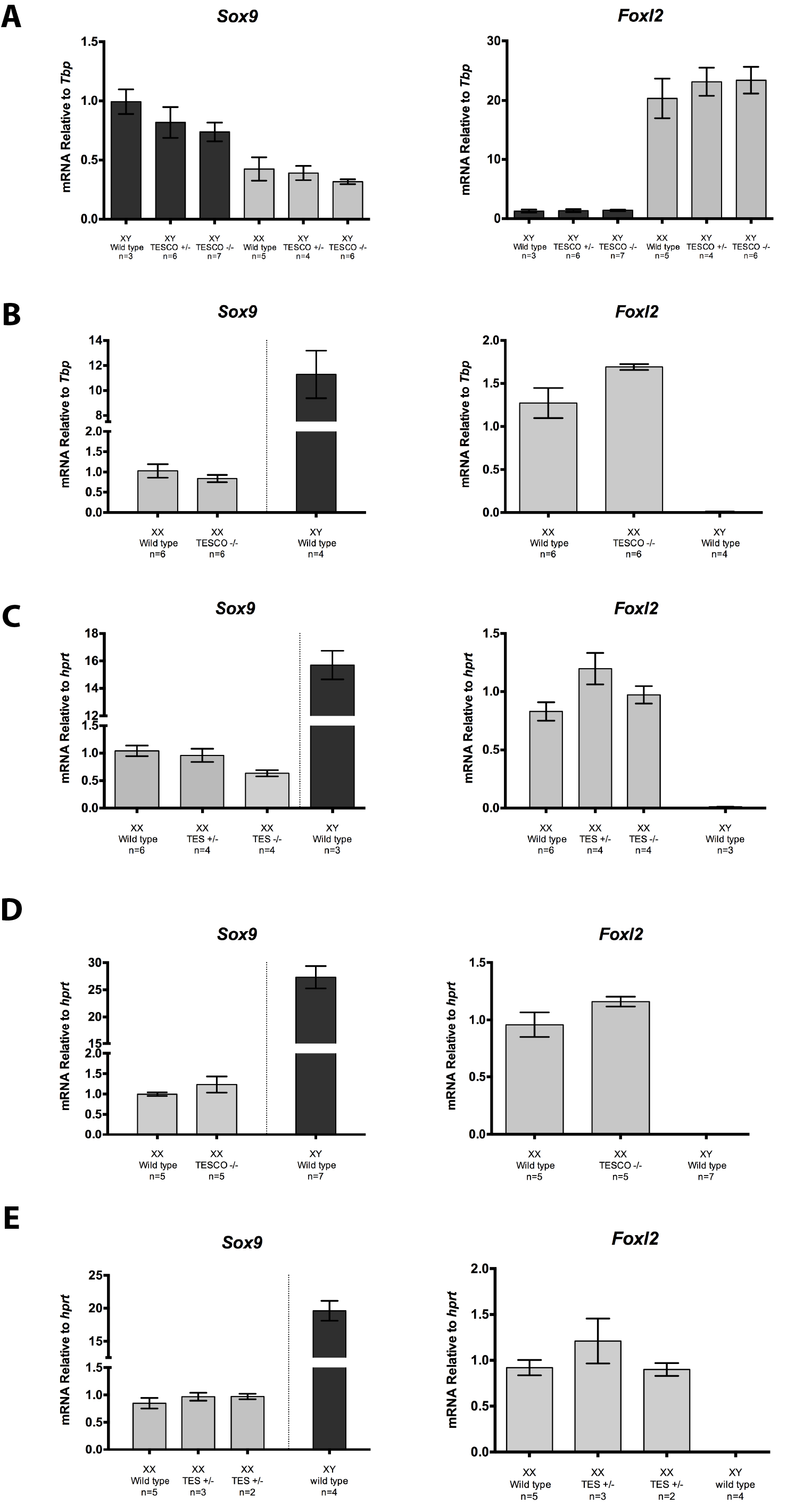

Supplement: S5 Fig — (A) Gene expression in XY TESCO deleted gonads-mesonephros pairs at 12.5 dpc (B) Gene expression in XX TESCO deleted gonads at 14.5 dpc (C) Gene expression in XX TES deleted gonads at 13.5 dpc (D) Gene expression in XX TESCO deleted gonads at 6 weeks (E) Gene expression in XX TES deleted gonads at 8 weeks. Data are presented as mean 2-ΔΔCt values normalized to Tbp/ Hprt. Sample size represents number of individuals and is indicated below each genotype. Error bars show SEM of 2-ΔΔCt values. P value is presented above the relevant bars (unpaired, two-tailed t-test on 2-ΔΔCt values). Dark grey bars: XY; light grey bars: XX. (TIF) [file pgen.1006520.s005.tif]
